# Real‐World Treatment Patterns Before and After Metastatic Castration–Resistant Prostate Cancer in Japan: Retrospective Analysis Using a Hospital‐Based, Multicenter Database

**DOI:** 10.1155/proc/9964591

**Published:** 2026-03-06

**Authors:** Masaki Shiota, Linghua Xu, Tomoyo Oguri, Masayuki Tanaka

**Affiliations:** ^1^ Department of Urology, Graduate School of Medical Sciences, Kyushu University, Fukuoka, Japan, kyushu-u.ac.jp; ^2^ Access and Value, Pfizer Japan Inc, Tokyo, Japan; ^3^ Genitourinary Cancer Team, Oncology Medical Affairs, Pfizer Japan Inc, Tokyo, Japan

**Keywords:** castration-sensitive prostate cancer, database analysis, Japanese prostate cancer patients, real-world, treatment sequence

## Abstract

**Background:**

Although treatment options for metastatic castration–resistant prostate cancer (mCRPC) have been increasing since 2014, the actual treatment sequences of each drug in clinical practice in Japan have only been reported in a limited number of patients and facilities. The primary objective of this database study was to investigate the general situation and the transition of prior and post‐mCRPC treatments.

**Methods:**

Using Medical Data Vision’s medical record database across Japan, patients diagnosed with mCRPC from January 2015 to December 2022 based on defined criteria were extracted. Treatment sequences before and after mCRPC diagnosis were analyzed.

**Results:**

Analysis of 4967 patients revealed that the most common pretreatment for mCRPC was classical vintage hormone therapy (77.1%), followed by androgen receptor signaling inhibitors (ARSI), including enzalutamide (7.8%) and abiraterone (7.4%). The most common treatments for first‐line mCRPC were enzalutamide (43.1%), abiraterone (28.3%), and docetaxel (10.7%). When the treatment prior to mCRPC was ARSI, docetaxel was the most common first‐line treatment for mCRPC, but another ARSI that had not been used as treatment prior to mCRPC was also selected as first‐line mCRPC treatment at a similar rate to docetaxel. Regarding annual changes, the proportion of vintage hormone therapy for mCRPC has been decreasing annually, and there has been a trend to replace it with ARSIs.

**Conclusion:**

In terms of the treatment sequence for mCRPC in Japan, vintage hormone therapy was the most common pretreatment for mCRPC, and ARSIs were the most common first‐line treatments for mCRPC.

## 1. Introduction

Globally, prostate cancer (PC) is the second most common cancer and the fifth leading cause of cancer‐related mortality among men, worldwide [1]. In Japan, PC is the most prevalent cancer among men, with 92,021 new cases reported in 2018 [2].

Recently, the treatment landscape for PC in Japan has changed greatly. Traditionally, PC was initially treated by medical castration with androgen deprivation therapy (ADT) alone; combined androgen blockade (CAB) with a combination of ADT and first‐generation antiandrogens (AAs); or estrogen therapy, which are collectively known as vintage hormone therapies. However, many patients eventually progress to the most advanced disease stage known as metastatic castration–resistant prostate cancer (mCRPC) [3].

Treatment options for mCRPC have advanced with the development of following treatment options, including docetaxel [4]; cabazitaxel [5]; novel androgen receptor signaling inhibitors (ARSIs), including enzalutamide [6, 7] and abiraterone [8, 9]; radium‐223 [10]; and the poly‐ADP ribose polymerase (PARP) inhibitor, olaparib [11]. More recently, treatment with docetaxel [12], enzalutamide [13–15], or abiraterone [16] has been used upfront in treating earlier stages of PC, metastatic castration–sensitive PC (mCSPC), and nonmetastatic CRPC (nmCRPC). Additionally, novel ARSI compounds, such as apalutamide [17, 18] and darolutamide [19, 20], have been approved for treating mCSPC and nmCRPC. The increasing diversity of treatment options for both pre‐ and post‐mCRPC treatment has complicated the selection of treatment sequences.

Despite numerous treatments, the prognosis of mCRPC remains poor. Enhancing prognosis via optimal therapy sequencing requires a comprehensive understanding of real‐world treatment sequences, especially as standard treatments and options rapidly evolve. For example, an analysis based on a physicians’ questionnaire survey revealed that abiraterone and enzalutamide were the most prescribed first‐line (1L) mCRPC treatments [21]. The Michinoku study [22], a multicenter retrospective study involving 252 patients aged ≥ 75 years, reported a difference in 1L mCRPC treatment between vintage hormone therapy or upfront therapies as prior treatments; when vintage hormone therapy was administered prior to the diagnosis of mCRPC, it remained the most commonly selected first‐line treatment for mCRPC. In contrast, when upfront therapies were used before mCRPC diagnosis, abiraterone was the most frequently chosen first‐line treatment. These studies were, however, limited by small sample sizes and the number of participating medical institutions, as well as the lack of year‐by‐year analysis. Evaluating large‐scale, real‐world treatment sequences before and after mCRPC diagnosis in Japan is essential to interpret temporal trends and inform clinical practice.

The primary objective of this database study was to investigate the current treatment sequence and its transition of pre‐ and post‐mCRPC in a Japanese real‐world setting, using a hospital‐based, multicenter database across Japan.

## 2. Materials and Methods

### 2.1. Study Design and Data Source

The study design was a descriptive, retrospective observational study focusing on patients diagnosed with mCRPC, using individual‐level anonymized healthcare claims data managed by Medical Data Vision Co., Ltd. (MDV). The MDV database, based on hospital claims and Diagnosis Procedure Combination (DPC) data, accommodates over 45 million patients from over 480 Japanese hospitals with 20+ beds (as of June 2024), with a significant portion being 65 years and older. It includes anonymized patient details, diagnoses, medical procedures, prescriptions, in/outpatient status, and lab data, covering both hospital stays and outpatient visits, including prescriptions post‐visit, unless there was a hospital transfer. Following Ethical Guidelines for Medical and Biological Research Involving Human Subjects, no institutional or ethics review was required because this analysis did not involve the collection, use, or transmittal of individually identifiable data (Chapter 1, Section 3, Subsection 1 on page 7 in the original Japanese version: https://www.mext.go.jp/lifescience/bioethics/files/pdf/n2373_01.pdf. English version: https://www.mext.go.jp/content/20250325-mxt_life-000035486-01.pdf).

**FIGURE 1:**
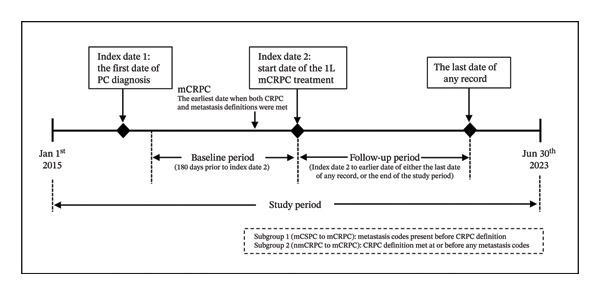
Study design diagram. The end of June 2023 was the most recent data at the time of study initiation. Abbreviations: CRPC, castration‐resistant prostate cancer; mCRPC, metastatic castration–resistant prostate cancer; nmCRPC, nonmetastatic castration–resistant prostate cancer; mCSPC, metastatic castration–sensitive prostate cancer; PC, prostate cancer.

The study design is shown in Figure 1. The study period was from January 01, 2015, to June 30, 2023, which includes mainstream mCRPC treatments approved in 2014, to the latest data available at the time of study initiation in September 2023. Two index dates were established in this study. Index date 1 represents the first date of PC diagnosis. Index date 2 was defined as the start date of the 1L mCRPC treatment. Index dates 1 and 2 were used to chronologically track the data and accurately determine the initiation and termination dates for pre‐mCRPC and first‐line mCRPC treatments, respectively. The baseline period was the 180‐day period before index date 2. The follow‐up period was from index date 2 to the last date of any record in the database or the end of the study period, whichever was earlier.

### 2.2. Patient Population

The study cohort comprised mCRPC patients who fulfilled all the inclusion criteria and none of the exclusion criteria below. Cancer diagnoses were established according to the International Classification of Diseases, 10th Edition (ICD‐10 codes).

#### 2.2.1. Inclusion Criteria

1. Confirmed PC diagnosis code from January 01, 2015, to June 30, 2023 (ICD‐10 codes in Supporting Data S1)
2. Age ≥ 20 at initial PC diagnosis
3. Satisfied at least one of the following CRPC definitions: CRPC diagnosis in the same or any subsequent months after initial PC diagnosis (ICD codes: C61 [8848040, 8851347]), or prostate‐specific antigen (PSA) level increase of > 1 ng/mL and > 25% from PSA nadir during ADT treatment.
4. Having any metastasis codes after the initial PC diagnosis (ICD codes in Supporting Data S2)
5. Records of medications prescribed as the 1L mCRPC treatment
6. Records of both a minimum 180‐day baseline period and a minimum 180‐day follow‐up period

#### 2.2.2. Exclusion Criteria

1. Any prior or concurrent primary malignancy or related metastases, other than PC, during the study period.
2. No initiation of 1L mCRPC treatment within 90 days after the earliest date when both CRPC and metastasis definitions were met.

### 2.3. Variables and Outcomes

Baseline characteristics included age, sex, duration from the index date 1 to 2, duration of the follow‐up period, and comorbidities observed during baseline periods. Comorbidities were summarized using ICD‐10 codes, with K70–K77, N00–N39/N99, I00–I99, E00–E90, and N40–42/N51.0 corresponding to liver, kidney, cardiovascular, metabolic, and prostatic diseases, respectively.

The list of PC medications in this study is shown in Supporting Data S3, and they are categorized as vintage hormone therapy, monotherapy, or combination therapy, as shown in Supporting Data S4. Estrogen therapy was included in the vintage hormone therapy category. Any medication that did not belong to any of these categories was classified as “Others.” The initiation date of a treatment line was the day on which a particular PC medication was first prescribed. Medications prescribed within 30 days of the initiation date were considered combination therapies. If the same medication was prescribed within 90 days from the last day of the prescribed medication quantity, it was considered that the same line had continued. The end date of a treatment line was the earlier date of either 90 days after the last prescription of the same medication or the date another medication was prescribed. The first treatment line post‐mCRPC definition was defined as 1L mCRPC therapy, with subsequent lines labeled sequentially (2L, 3L, etc.). The treatment just before the 1L mCRPC therapy was defined as pre‐mCRPC treatment.

The treatment sequence from pre‐ and post‐mCRPC treatment, the annual number and percentages of patients prescribed with each pre‐mCRPC and 1L mCRPC treatment, and the duration of each treatment at pre‐ and 1L‐mCRPC treatment were evaluated.

### 2.4. Data Sets and Statistical Analysis

The full set includes all patients extracted by the inclusion and exclusion criteria. Cases with metastasis codes before meeting the CRPC definition were marked as transitioning from mCSPC to mCRPC, or Subgroup 1. Cases who met CRPC definition before any records of metastasis codes were marked as transitioning from nmCRPC to mCRPC, or Subgroup 2. Cases simultaneously meeting both CRPC and metastasis definitions were included in Subgroup 2. The full set and the subgroups were analyzed for the treatment sequences from pre‐mCRPC to 1L mCRPC treatment and annual trends, which were specified as primary outcomes. The remaining analyses, including treatment sequence from 1L to 5L and each treatment duration in pre‐mCRPC and 1L mCRPC treatment, were conducted only on the full set.

Continuous data were analyzed as the number and percentages of patients along with descriptive statistics (mean, standard deviation, median, minimum and maximum, first and third quartiles). Categorical data were reported as frequency (*n*, %). The treatment sequence was presented as frequencies and percentages in Sankey diagrams. Data extraction and analysis were performed using Statistical Analysis Software (SAS Institute, Cary, NC, USA, Version 9.4) and R Version 4.2.0.

## 3. Results

**FIGURE 2:**
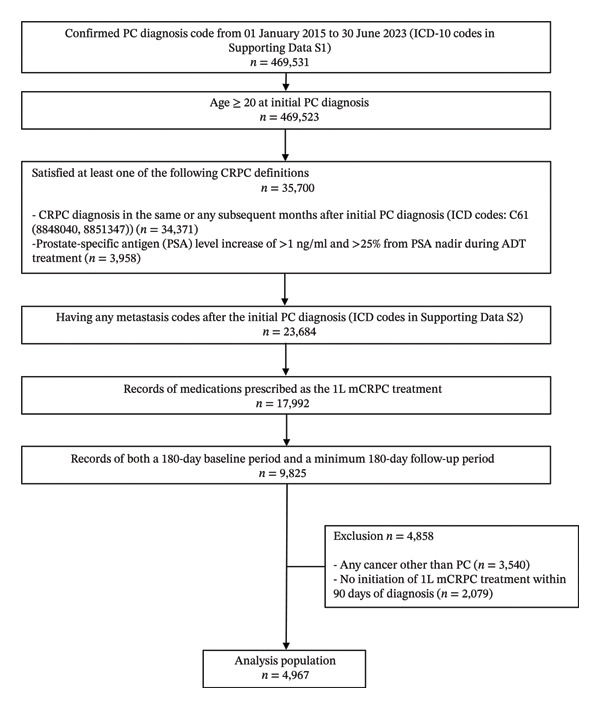
Patient disposition. Abbreviations: ADT, androgen deprivation therapy; CRPC, castration‐resistant prostate cancer; mCRPC, metastatic castration–resistant prostate cancer; PC, prostate cancer.

A total of 469,531 patients were identified with a confirmed PC diagnosis from January 01, 2015, to June 30, 2023 (Figure 2). Among these patients, 9825 patients were selected after applying the inclusion criteria. Regarding the third inclusion criteria for CRPC definition, PSA levels were only available for 10.1% of all patients aged ≥ 20 (47,397/469,523), because laboratory parameters are typically available only for a subset of patients in this database, as it is based on hospital claims data; the majority of CRPC patients were consequently identified by CRPC diagnosis code rather than by their PSA level (34,371 vs. 3958). The final full analysis set comprised 4967 patients after applying the exclusion criteria (Figure 2).

Patient demographics are shown in Table 1. The mean ages (±SD) at index dates 1 and 2 were 74.1 ± 7.8 years and 76.0 ± 7.7 years, respectively. The mean duration (±SD) from the date of mCRPC to index date 2 was 6.7 ± 17.6 days. The rates of each comorbidity were as follows: Liver disease was observed in 14.5%, kidney disease in 42.3%, cardiovascular disease in 37.6%, metabolic disease in 51.8%, and prostatic disease in 54.4%. In addition, the mean duration of the follow‐up period (±SD) was 784.9 ± 514.0 days (data not shown).

**TABLE 1:** Demographics and baseline characteristics.

Item	Total (*n* = 4967)
Age at index date 1 (years)	
Mean ± SD	74.1 ± 7.8
Median (Q1, Q3)	74.0 (69.0, 80.0)
Age at index date 2 (years)	
Mean ± SD	76.0 ± 7.7
Median (Q1, Q3)	76.0 (71.0, 82.0)
Age range at index date 2	
≤ 49, *n* (%)	8 (0.2)
50–59, *n* (%)	107 (2.2)
60–69, *n* (%)	858 (17.3)
70–79, *n* (%)	2282 (45.9)
80–89, *n* (%)	1575 (31.7)
≥ 90, *n* (%)	137 (2.8)
Sex	
Male, *n* (%)	4967 (100.0)
Female, *n* (%)	0 (0.0)
Duration from the date of mCRPC to index date 2 (days)^a^	
Mean ± SD	6.7 ± 17.6
Median (Q1, Q3)	0.0 (0.0, 0.0)
Comorbidities	
Liver disease, *n* (%)	721 (14.5)
Kidney disease, *n* (%)	2101 (42.3)
Cardiovascular disease, *n* (%)	1870 (37.6)
Metabolic disease, *n* (%)	2572 (51.8)
Prostatic disease, *n* (%)	2704 (54.4)
Metastatic site	
Bone, *n* (%)	4338 (87.3)
Lymph node, *n* (%)	1077 (21.7)
Lung, *n* (%)	208 (4.2)
Liver, *n* (%)	53 (1.1)

Abbreviations: mCRPC, metastatic castration–resistant prostate cancer; mCSPC, metastatic castration–sensitive prostate cancer; nmCRPC, nonmetastatic castration–resistant prostate cancer; Q1, first quartile; Q3, third quartile; SD, standard deviation.

^a^The dates of CRPC diagnoses by ICD‐10 codes were made on the last day of each month. If 1L or treatment was started in the same month, the duration from mCRPC to the index date 2 was calculated as “0.”

Treatment sequence from pre‐ to 1L‐mCRPC treatment was analyzed (Figure 3(a)). The most common pre‐mCRPC treatment was vintage hormone therapy, accounting for 77.1% of the total, followed by enzalutamide and abiraterone at 7.8% and 7.4%, respectively. In 1L mCRPC treatment, enzalutamide (43.1%) and abiraterone (28.3%) were the most frequently used medications, followed by docetaxel (10.7%) and radium‐223 (4.3%). For each pre‐mCRPC treatment with vintage hormone therapy, enzalutamide, or abiraterone, their subsequent treatments at 1L mCRPC are separately shown in Figures 3(b)–3(d). Notably, there was a preferential switch from vintage hormone therapy to enzalutamide (52.2%) or abiraterone (31.3%) (Figure 3(b)). Conversely, when the pre‐mCRPC treatment was enzalutamide (Figure 3(c)) or abiraterone (Figure 3(d)), 1L mCRPC treatments were mainly switched to docetaxel: 31.3% from enzalutamide and 29.6% from abiraterone. Although sequencing therapy among ARSIs is not recommended in reports such as the Advanced Prostate Cancer Consensus Conference [23], switching between both ARSIs was also observed: 23.4% switched from enzalutamide to abiraterone and 29.0% vice versa.

FIGURE 3(a) Treatment sequence from pre‐mCRPC to 1L treatment (overall population). (b) 1L treatment of patients in which pre‐mCRPC treatment was vintage hormone therapy. (c) 1L treatment of patients in which pre‐mCRPC treatment was enzalutamide. (d) 1L treatment of patients in which pre‐mCRPC treatment was abiraterone. Notes: Vintage hormone therapy group comprises treatment with antiandrogens (AAs); androgen deprivation therapy (ADT); combined androgen blockade (CAB); or estrogen therapy.

**Figure:**
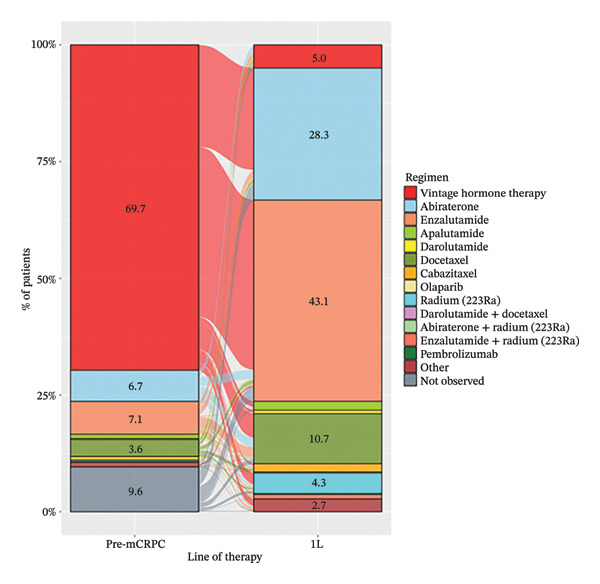
(a)

**Figure:**
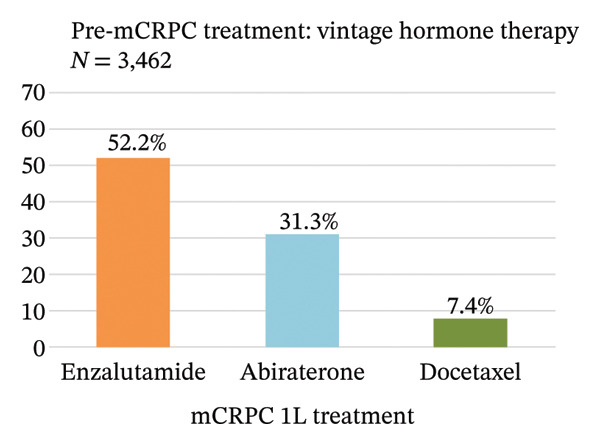
(b)

**Figure:**
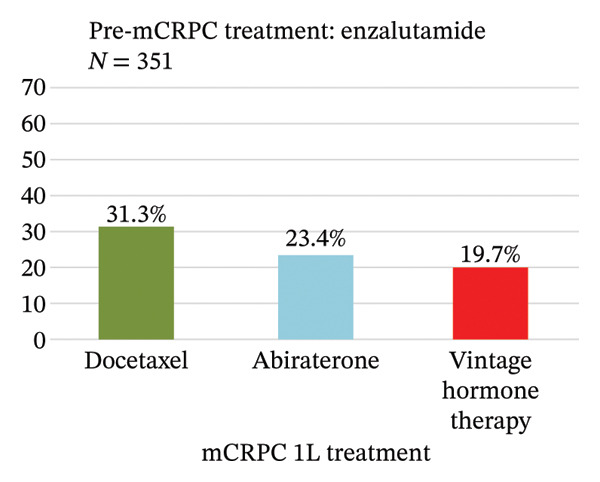
(c)

**Figure:**
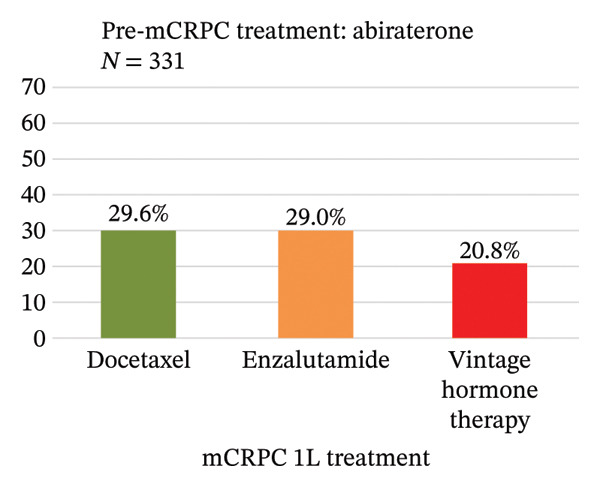
(d)

**FIGURE 4:**
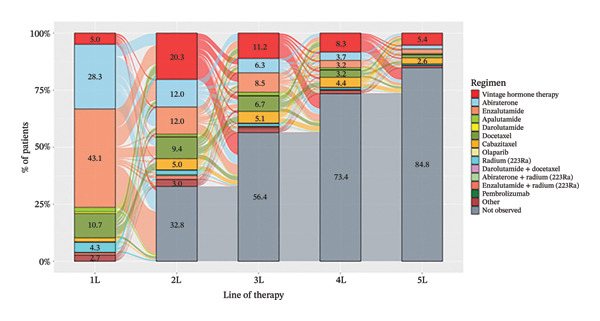
Treatment sequence from 1L to 5L (overall population). 1L mCRPC therapy is the first treatment line initiated after mCRPC definition, followed by 2L, 3L, 4L, and 5L, as each time when the treatment was switched to a new line. Notes: Vintage hormone therapy group comprises treatment with antiandrogens (AAs); androgen deprivation therapy (ADT); combined androgen blockade (CAB); or estrogen therapy.

Subsequent treatments after 1L were also evaluated until 5L treatments (Figure 4). The number of patients for whom no therapy was observed actually increased from 2L to 5L. In 2L treatment, the proportions of patients using enzalutamide (17.8%), abiraterone (17.9%), and docetaxel (14.0%) were approximately equivalent, and this trend persisted up to 5L treatment. Among 1L and 2L treatments, switches from enzalutamide to abiraterone, or vice versa, were more commonly observed than from each treatment to docetaxel. The 2L treatment of docetaxel was the most commonly switched from enzalutamide or abiraterone. Following 2L, cabazitaxel use increased relative to 1L and represented the predominant switch from docetaxel; in 1L through 5L, the number of patients receiving cabazitaxel was 88 (1.8%), 246 (5.0%), 254 (5.1%), 219 (4.4%), and 131 (2.6%), respectively, with prior docetaxel exposure observed in 33/88 (34.1%), 159/246 (64.6%), 127/254 (50.0%), 104/219 (47.5%), and 49/131 (37.4%) of these patients. The use of olaparib was consistently low across all treatment lines.

Annual trends of each treatment at pre‐mCRPC and 1L treatments were analyzed. At pre‐mCRPC treatment, the percentage use of vintage hormone therapies decreased annually from 90.1% in 2015 to 41.8% in 2022 (Figure 5(a)). Meanwhile, the use of ARSIs, including abiraterone, enzalutamide, apalutamide, and darolutamide, increased every year. Regarding 1L mCRPC treatment, annual changes were not as pronounced as in pre‐mCRPC treatment, with the use of each therapy remaining relatively constant from 2015 to 2022 (Figure 5(b)). During this period, enzalutamide and abiraterone were the most common therapies, used by 55.0%–41.8% and 27.8%–24.2% of patients, respectively. Docetaxel and radium‐223 monotherapy were also used by a substantial percentage of patients.

FIGURE 5Annual treatment trends of pre‐mCRPC and 1L treatment. (a) Pre‐mCRPC treatment. (b) 1L mCRPC treatment. Notes: Vintage hormone therapy group comprises treatment with antiandrogens (AAs); androgen deprivation therapy (ADT); combined androgen blockade (CAB); or estrogen therapy.

**Figure:**
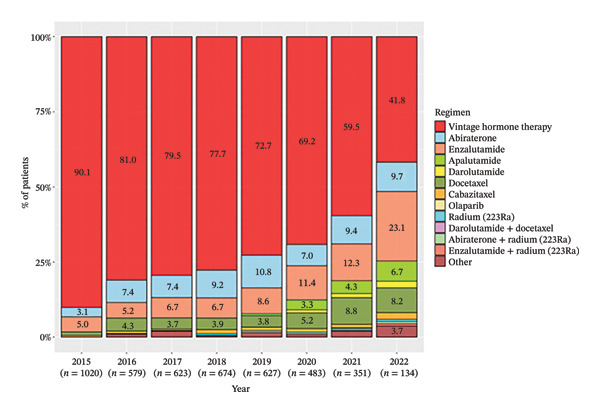
(a)

**Figure:**
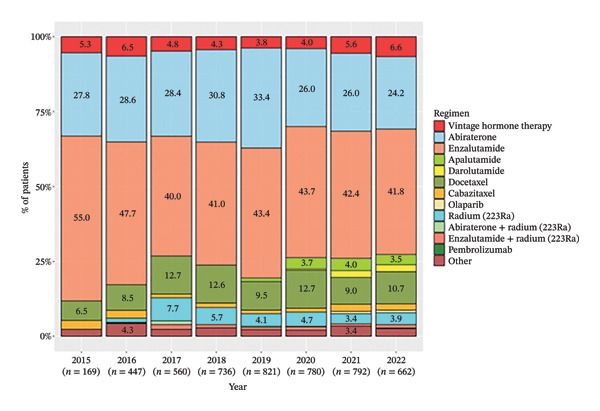
(b)

The durations of each treatment were also analyzed. Regarding pre‐mCRPC treatment, the three most frequent treatments were vintage hormone therapy, enzalutamide, and abiraterone, with mean durations of 594.7, 298.3, and 239.6 days, respectively (Supporting Data S5A). In 1L mCRPC treatment, the 4 most common treatments were enzalutamide, abiraterone, apalutamide, and darolutamide with mean durations of 506.2, 427.0, 397.4, and 323.8 days, respectively (Supporting Data S5B).

Subgroup analyses were performed for Subgroup 1 (mCSPC to mCRPC) and 2 (nmCRPC to mCRPC). The overall patient demographics of both groups were similar to the total analysis set (Supporting Data S6). Regarding treatment sequence from pre‐ to 1L‐mCRPC treatment, in Subgroup 1 (Supporting Data S7A), vintage hormone therapy was the dominant pre‐mCRPC treatment (85.7%), and the most common 1L mCRPC therapies were enzalutamide (49.2%), abiraterone (30.3%), and docetaxel (7.8%). In Subgroup 2 (Supporting Data S7B), the major pre‐mCRPC treatments were vintage hormone therapy (44.9%), enzalutamide (22.3%), and abiraterone (17.8%) in order of most common. For 1L mCRPC therapy in Subgroup 2, enzalutamide, abiraterone, and docetaxel were used with similar proportions at 25.3%, 22.1%, and 19.3%, respectively. In terms of annual trends of pre‐ and 1L‐mCRPC treatments, the percentage use of vintage hormone therapy in Subgroup 1 decreased from 2015 to 2022 from 93.1% to 54.2%, being replaced by ARSIs including abiraterone, enzalutamide, and apalutamide (Supporting Data S8A). The major 1L mCRPC treatments in this subgroup were enzalutamide and abiraterone from 2015 to 2022 (Supporting Data S8B). Regarding the pre‐mCRPC treatment of Subgroup 2, the percentage use of both ARSIs and docetaxel moderately increased every year (Supporting Data S8C). In this group, the majority of 1L mCRPC treatments in 2015 and 2016 were abiraterone and enzalutamide, while some parts of them were replaced by docetaxel, radium‐223, and apalutamide after 2017 (Supporting Data S8D).

## 4. Discussion

This study presents the first comprehensive, large‐scale examination of the evolution and current state of pre‐ and post‐mCRPC treatment in Japan, based on real‐world data from a hospital‐based, multicenter database across Japan. Following mCRPC diagnosis, ARSIs, particularly abiraterone and enzalutamide, were the primary therapeutic medications in 1L. This aligns with the NCCN guideline, Version 4.2023, the most current guideline relevant to the study period, which mentions these medications are preferred 1L treatments for mCRPC [24]. In addition, a survey‐based analysis among Japanese physicians demonstrates that abiraterone and enzalutamide were the most commonly selected first‐line treatments for mCRPC [21]. Meanwhile, in the Michinoku multicenter retrospective study of 146 patients who had received ADT as pretreatment for mCRPC reported in 2020, docetaxel and ARSIs were used as 1L mCRPC therapies in 53% and 47% of patients, respectively [25]. However, the Michinoku study included periods before 2014 when ARSIs were not approved in Japan, which could have resulted in a higher docetaxel use.

Although the annual use of vintage hormone therapies is being replaced by ARSIs such as abiraterone and enzalutamide, vintage hormone therapy remains the most prevalent pre‐mCRPC treatment. Data in Japan on 125 patients reported by 52 physicians selected from a physicians’ panel in 2020 showed that nearly 80% of patients with mCSPC were treated with ADT/CAB [26]. Moreover, Japanese urologists are reported to show a preferential tendency to prescribe ADT monotherapy compared to other countries [27]. It is interesting to note that vintage hormone therapy still accounted for the majority of cases. However, these results may also include some biases. For example, this study selected patients who were defined as mCRPC, resulting in the exclusion of those who did not progress to mCRPC due to effective treatments. Since upfront treatment with ARSIs usually improves time to CRPC compared to that with vintage hormone therapy, the proportion of patients where ARSIs were used for pre‐mCRPC may have been underestimated in this study. This selection bias should be considered for the interpretation of the proportion of each treatment.

Patients treated by vintage hormone therapies predominantly received ARSIs for 1L mCRPC. In comparison, when ARSIs were used upfront as pre‐mCRPC therapies, the use of docetaxel was higher in 1L mCRPC, while there was also ARSI switching at the same proportion. Since cross‐resistance has been reported among ARSI therapies [28], docetaxel may therefore be selected preferentially for patients already treated by ARSIs. Moreover, as reported in the Michinoku study, mCRPC patients treated by ARSIs switched to unused ARSIs at the same rate as to docetaxel [25]. The switch between ARSIs may also be chosen in preference to docetaxel, according to patients’ general backgrounds, including elderly age, tolerability to adverse events, and preferences.

Regarding treatment after 1L mCRPC, the percentage use of cabazitaxel increased after 2L, and it was mainly switched from docetaxel in each line. In the NCCN guideline [24], cabazitaxel is recommended as a preferred treatment for mCRPC after docetaxel relapse. Our results confirmed that, following the recommendation, cabazitaxel is the treatment of choice postdocetaxel in real‐world clinical practice.

Despite the approval of olaparib for *BRCA*‐positive mCRPC in 2020 [11], its use in mCRPC 1L–5L was low. The ZENSHIN study recently reported a *BRCA*‐positive rate of 13.3% among Japanese mCRPC patients [29]. Also, in 2020, the genetic testing rate among mCRPC patients in Japan was only 2.9% [30]. The low positive rate and testing coverage may explain the underuse of PARP inhibitors. However, with the recent approval of the new PARP inhibitor, talazoparib, for treating *BRCA*‐positive mCRPC in Japan, the use of such medications may increase due to the growing availability of PARP therapies.

There are some possible limitations in this study. Although mCRPC, mCSPC, and nmCRPC were defined based on diagnosis codes and PSA values, validation of their accuracy was not conducted. Laboratory parameters, including PSA, are typically available only for a subset of patients in this database, as the MDV database is based on hospital claims data. Confounding factors, including cancer severity, patient condition, and economic status, which could influence treatment choice, cannot be considered in the database used. Any reasons for treatment change or termination, including adverse events, drug inefficacy, death, or transfers, cannot be captured. The MDV database is hospital‐based and therefore lacks data from smaller hospitals and clinics not under the DPC system, and also does not allow for patient traceability across different hospitals, which may limit external generalizability and introduce selection bias. Additionally, as the MDV database enrolls patients annually, there might be yearly variations in enrollment sites and patient backgrounds.

## 5. Conclusion

This study found that while vintage hormone therapy was the dominant pre‐mCRPC treatment, its use is declining yearly. ARSIs, particularly abiraterone and enzalutamide, have become the primary mCRPC 1L treatments after their launch in 2014. These results are considered to be influenced by the high efficacy of treatments for mCRPC, both before and after its onset, which has been demonstrated in recent years. ARSIs were mainly used as 1L treatments in patients treated with vintage hormone therapy at pre‐mCRPC. However, if an ARSI medication was used upfront for pre‐mCRPC treatment, docetaxel was more likely in 1L, while ARSI switching also occurred. Due to cross‐resistance among ARSIs, treatment with another ARSI after upfront ARSI therapy is generally not recommended; however, depending on patient background or preferences, there may be situations in which the use of an alternative ARSI is unavoidable. There was an increasing use of taxanes post‐1L mCRPC treatment. The underuse of PARP inhibitors may be attributed to the low *BRCA*‐positive rate and limited genetic test coverage. As pre‐ and post‐mCRPC treatment options change annually, further studies are necessary to understand the treatment landscape for future discussion about the prognosis of mCRPC.

## Funding

This work was sponsored and funded by Pfizer Japan Inc., which played a specific role in the conceptualization, design, data collection, analysis, decision to publish, and preparation of the manuscript. Additionally, Pfizer Japan Inc. contracted and funded Medical Data Vision Co., Ltd. (Tokyo, Japan) for data provision and extraction services, and Clinical Study Support Inc. (Nagoya, Japan) for statistical analysis, medical writing, and editorial services.

## Ethics Statement

The study was conducted in accordance with legal and regulatory requirements, as well as with scientific purpose, value, and rigor and followed accepted research practices described in Guidelines for Good Pharmacoepidemiology Practices issued by the International Society for Pharmacoepidemiology, Good Practices for Outcomes Research issued by the International Society for Pharmacoeconomics and Outcomes Research, and International Ethical Guidelines for Epidemiological Studies issued by the Council for International Organizations of Medical Sciences.

## Consent

As this study involved anonymized structured data, which, according to applicable legal requirements, do not contain data subject to privacy laws, obtaining informed consent from patients was not required.

## Conflicts of Interest

In the previous 2 years, Masaki Shiota received honoraria from Janssen Pharmaceutical, AstraZeneca, Astellas Pharma, Sanofi, and Bayer and research funding support from Astellas Pharma. Linghua Xu, Tomoyo Oguri, and Masayuki Tanaka are employees of Pfizer Japan Inc. Masaki Shiota, Linghua Xu, Tomoyo Oguri, and Masayuki Tanaka contributed to the study design, interpretation of data, writing of the report, and the decision to submit the report for publication. Data collection and analysis were outsourced to Clinical Study Support, Inc., Japan.

## Supporting Information

Supporting Data S1: PC disease code. List of ICD‐10 codes used for prostate cancer diagnoses.

Supporting Data S2: Metastasis disease codes. List of ICD‐10 codes used for prostate cancer metastases.

Supporting Data S3: PC medication list. List of medications used to treat prostate cancer analyzed in this study.

Supporting Data S4: Regimen list. Description of treatment regimens for prostate cancer analyzed in this study.

Supporting Data S5A: Each treatment duration: pre‐mCRPC treatment (days). The treatment duration, in days, of each treatment regimen in pre‐mCRPC treatment.

Supporting Data S5B: Each treatment duration: 1L mCRPC treatment (days). The treatment duration, in days, of each treatment regimen in first‐line mCRPC treatment.

Supporting Data S6: Demographics and baseline characteristics, total population, and subgroups. The demographics and baseline characteristics of patients included in the total analysis population and the subgroup analyses.

Supporting Data S7: Treatment sequence before and after mCRPC (subgroup analysis).

S7A Subgroup 1: Sankey diagram of the percentages of patients taking each treatment regimen at pre‐mCRPC and first‐line mCRPC treatment in Subgroup 1.

S7B Subgroup 2: Sankey diagram of the percentages of patients taking each treatment regimen at pre‐mCRPC and first‐line mCRPC treatment in Subgroup 2.

Supporting Data S8: Annual trends of pre‐ and 1L‐mCRPC treatment (subgroup analysis).

S8A: Pre‐mCRPC treatment (Subgroup 1): The percentages of patients taking each treatment regimen for pre‐mCRPC treatment in Subgroup 1 in each year during 2015–2022.

S8B, S8B: 1L mCRPC treatment (Subgroup 1): The percentages of patients taking each treatment regimen for 1L mCRPC treatment in Subgroup 1 in each year during 2015–2022.

S8C: Pre‐mCRPC treatment (Subgroup 2): The percentages of patients taking each treatment regimen for pre‐mCRPC treatment in Subgroup 2 in each year during 2015–2022.

S8D, S8D: 1L mCRPC treatment (Subgroup 2): The percentages of patients taking each treatment regimen for 1L mCRPC treatment in Subgroup 2 in each year during 2015–2022.

## Supporting information


**Supporting Information** Additional supporting information can be found online in the Supporting Information section.

## Data Availability

The data that support the findings of this study are available from Medical Data Vision Co., Ltd. (MDV) (Tokyo, Japan), but restrictions apply to the availability of these data, which were used under license for the current study, and so are not publicly available. Data are, however, available from the authors upon reasonable request and with permission of MDV.
