# Supplementary material for: Real‐World Treatment Patterns Before and After Metastatic Castration–Resistant Prostate Cancer in Japan: Retrospective Analysis Using a Hospital‐Based, Multicenter Database
Source: Prostate Cancer. 2026 Mar 6;2026:9964591. doi: 10.1155/proc/9964591 (PMC12964489; doi:10.1155/proc/9964591)
Supplement: Supplementary file 1 — Supporting Information Additional supporting information can be found online in the Supporting Information section. [file PROC-2026-9964591-s001.pdf]

**Supplementary Data S1: PC disease code**

| Disease name | ICD10 code | Disease code |
|---|---|---|
| castration-resistant prostate cancer | C61 | 8848040 |
| localized prostate cancer | C61 | 8848043 |
| progressive prostate cancer | C61 | 8848066 |
| prostate cancer | C61 | 1859003 |
| prostate cancer recurrence | C61 | 8848074 |
| prostate small cell carcinoma | C61 | 8845980 |
| prostate neuroendocrine carcinoma | C61 | 8842789 |
| castration-resistant prostate cancer with metastasis | C61 | 8851347 |
| prostate cancer bone metastasis | C795 | 8842788 |
| metastatic prostate cancer | C798 | 8850888 |

Notes: PC, prostate cancer.

**Supplementary Data S2: Metastasis disease codes**

| Disease name | ICD 10 code | Disease code |
|---|---|---|
| choroidal metastatic cancer | C794 | 1906005 |
| cervical lymph node metastasis | C770 | 1960005 |
| lymph node metastasis | C779 | 1969001 |
| metastatic lung tumors | C780 | 1970006 |
| metastatic mediastinal tumor | C781 | 1971002 |
| metastatic pleural tumors | C782 | 1972003 |
| metastatic liver tumors | C787 | 1977006 |
| metastatic renal tumors | C790 | 1980004 |
| metastatic skin tumors | C792 | 1982004 |
| supratentrial and subtentrial metastatic tumors | C793 | 1983001 |
| occipital metastatic tumors | C793 | 1983003 |
| frontal metastatic tumor | C793 | 1983005 |
| temporal metastatic tumor | C793 | 1983006 |
| deep cerebral metastatic tumor | C793 | 1983009 |
| metastatic spinal cord tumors | C794 | 1983014 |
| metastatic brain tumors | C793 | 1983019 |
| metastatic spinal cord epidural tumor | C794 | 1984001 |
| metastatic spinal intradural extramedullary tumor | C794 | 1984002 |
| metastatic bone cancer | C795 | 1985007 |
| pelvic metastasis | C795 | 1985008 |
| metastatic bone tumor | C795 | 1985023 |
| metastatic skull tumor | C795 | 1985024 |
| metastatic neck tumors | C798 | 1988009 |
| generalized metastatic cancer | C799 | 1990004 |
| multiple cancer metastasis | C799 | 1990009 |
| metastatic tumors | C799 | 1991019 |
| metastatic cardiac tumors | C798 | 2398068 |
| metastatic supraclavicular tumors | C770 | 8834006 |
| spinal metastasis | C795 | 8836022 |
| groin lymph node metastasis | C774 | 8836801 |
| metastatic femoral bone tumor | C795 | 8837313 |
| metastatic retroperitoneal tumors | C786 | 8837926 |
| metastatic gastrointestinal tumors | C788 | 8837928 |
| metastatic small intestinal tumor | C784 | 8837929 |
| metastatic pancreatic tumor | C788 | 8837930 |
| metastatic colorectal tumor | C785 | 8837931 |

| | | |
|---|---|---|
| metastatic vaginal tumor | C798 | 8837932 |
| metastatic splenic tumor | C788 | 8837933 |
| metastatic adrenal tumor | C797 | 8837934 |
| abdominal lymph node metastasis | C772 | 8839656 |
| peritoneal metastasis | C786 | 8839765 |
| costal metastasis | C795 | 8841259 |
| axillary lymph node metastasis | C773 | 8842679 |
| supraclavicular lymph node metastasis | C770 | 8842736 |
| mediastinal lymph node metastasis | C771 | 8842764 |
| prostate cancer bone metastasis | C795 | 8842788 |
| metastatic rectal tumor | C785 | 8842810 |
| thoracic vertebral metastasis | C795 | 8842905 |
| lumbar vertebral metastasis | C795 | 8843015 |
| metastatic chest wall tumor | C798 | 8843435 |
| bone marrow metastasis | C795 | 8844442 |
| bronchial lymph node metastasis | C771 | 8844689 |
| pelvic lymph node metastasis | C775 | 8844873 |
| multiple lymph node metastasis | C778 | 8845584 |
| retroperitoneal lymph node metastasis | C772 | 8845660 |
| para-aortic lymph node metastasis | C772 | 8845706 |
| iliac lymph node metastasis | C775 | 8845709 |
| hilar lymph node metastasis | C771 | 8845718 |
| splenic hilar lymph node metastasis | C772 | 8845728 |
| cetastatic tracheal tumor | C783 | 8847055 |
| metastatic abdominal wall tumor | C792 | 8847798 |
| cervical spine metastasis | C795 | 8848241 |
| diaphragmatic metastasis | C798 | 8848470 |
| perigastric lymph node metastasis | C772 | 8848689 |
| hepatic portal lymph node metastasis | C772 | 8848695 |
| parasternal lymph node metastasis | C771 | 8848703 |
| mesenteric lymph node metastasis | C772 | 8848748 |
| metastatic soft tissue tumors | C798 | 8850285 |
| metastatic gastric tumor | C788 | 8850885 |
| metastatic prostate cancer | C798 | 8850888 |
| subclavian lymph node metastasis | C773 | 8850990 |
| systemic lymph node metastasis | C778 | 8851008 |
| multiple lymph node metastasis | C778 | 8851011 |

| | | |
|---|---|---|
| castration-resistant prostate cancer with metastasis | C61 | 8851347 |

**Supplementary Data S3: PC Medication list**

| General name |
|---|
| Leuprorelin Acetate |
| Goserelin Acetate |
| Degarelix Acetate |
| Estramustine Phosphate Sodium Hydrate |
| Ethinylestradiol |
| Chlormadinone Acetate |
| Bicalutamide |
| Flutamide |
| Apalutamide |
| Abiraterone Acetate |
| Ifosfamide |
| Enzalutamide |
| Olaparib |
| Cabazitaxel Acetonate |
| Cisplatin |
| Darolutamide |
| Tegafur/Uracil |
| Docetaxel |
| Radium(223Ra) Chloride |
| Pembrolizumab |

Notes: PC, prostate cancer.

**Supplementary Data S4: Regimen list**

| Category | Regimen name | Drug list |
|---|---|---|
| Vintage hormone therapy | ADT/CAB | Leuprorelin<br>Goserelin<br>Degarelix<br>Chlormadinone + ADT<br>Bicalutamide + ADT<br>Flutamide + ADT<br>Estramustine + ADT<br>Ethinylestradiol + ADT |
| | AA | Bicalutamide, Flutamide, or Chlormadinone (without ADT) |
| | estrogen therapy | Estramustine or Ethinylestradiol (without ADT) |
| Monotherapy | Abiraterone | Abiraterone ± ADT/CAB ± estrogen therapy |
| | Enzalutamide | Enzalutamide ± ADT/CAB ± estrogen therapy |
| | Apalutamide | Apalutamide ± ADT/CAB ± estrogen therapy |
| | Darolutamide | Darolutamide ± ADT/CAB ± estrogen therapy |
| | Docetaxel | Docetaxel ±ADT/CAB ± estrogen therapy |
| | Cabazitaxel | Cabazitaxel ± ADT/CAB ± estrogen therapy |
| | Olaparib | Olaparib ±ADT/CAB ±estrogen therapy |
| | Radium(223Ra) | Radium(223Ra) ± ADT/CAB ± estrogen therapy |
| | Pembrolizumab | Pembrolizumab ± ADT/CAB ± estrogen therapy |
| | Darolutamide + Docetaxel | Darolutamide + Docetaxel,<br>or Darolutamide + Docetaxel± ADT/CAB ± estrogen therapy |
| | Abiraterone + Radium(223Ra) | Abiraterone + Radium(223Ra), or Abiraterone + Radium(223Ra) ±ADT/CAB ± estrogen therapy |
| Combination therapy | Enzalutamide + Radium(223Ra) | Enzalutamide + Radium(223Ra), or Enzalutamide + Radium(223Ra) ±ADT/CAB ±estrogen therapy |
| | Olaparib + Radium(223Ra) | Olaparib + Radium(223Ra), or Olaparib + Radium(223Ra) ±ADT/CAB ± estrogen therapy |

Notes: AA, antiandrogens; ADT, androgen deprivation therapy; CAB, combined androgen-blockage.

**Supplementary data S5A: Each treatment duration: pre-mCRPC treatment (Days)**

| Pre-mCRPC treatment regimen | N (%) | Mean (SD) | Max | Q3 | Median | Q1 | Min |
|---|---|---|---|---|---|---|---|
| Total | 4967 (100.0) | 513.6 (490.2) | 2826 | 669.0 | 350.0 | 181.0 | 1 |
| Vintage hormone therapy | 3462 (69.7) | 594.7 (513.2) | 2826 | 784.0 | 430.5 | 238.0 | 1 |
| Abiraterone | 331 (6.7) | 239.6 (251.5) | 2023 | 315.0 | 142.0 | 84.0 | 7 |
| Enzalutamide | 351 (7.1) | 298.3 (319.1) | 1940 | 371.0 | 176.0 | 96.0 | 20 |
| Apalutamide | 44 (0.9) | 205.1 (177.3) | 707 | 269.5 | 137.5 | 76.5 | 2 |
| Darolutamide | 13 (0.3) | 205.9 (141.4) | 496 | 240.0 | 196.0 | 109.0 | 14 |
| Docetaxel | 177 (3.6) | 200.0 (148.3) | 931 | 252.0 | 155.0 | 105.0 | 28 |
| Cabazitaxel | 42 (0.8) | 225.2 (225.4) | 1246 | 273.0 | 137.0 | 91.0 | 41 |
| Olaparib | 1 (0.0) | 421.0 (-) | 421 | 421.0 | 421.0 | 421.0 | 421 |
| Radium(223Ra) | 16 (0.3) | 165.4 (39.8) | 215 | 192.5 | 171.5 | 151.0 | 63 |
| Darolutamide + Docetaxel | 1 (0.0) | 405.0 (-) | 405 | 405.0 | 405.0 | 405.0 | 405 |
| Abiraterone + Radium(223Ra) | 2 (0.0) | 53.5 (43.1) | 84 | 84.0 | 53.5 | 23.0 | 23 |
| Enzalutamide + Radium(223Ra) | 2 (0.0) | 67.5 (23.3) | 84 | 84.0 | 67.5 | 51.0 | 51 |
| Other | 49 (1.0) | 72.8 (136.1) | 763 | 35.0 | 28.0 | 21.0 | 1 |

Aggregated pre-mCRPC treatment regimens, regardless of whether a regimen is prescribed after 1L. Pre-mCRPC treatment is the last treatment line just before index date. 1L mCRPC therapy is the first treatment line initiated after index date.

Notes: Vintage hormone therapy group comprises: treatment with antiandrogens (AA); androgen deprivation therapy (ADT); combined androgen blockade (CAB); or estrogen therapy ; mCRPC, metastatic castration-resistant prostate cancer; SD, standard deviation.

**Supplementary data S5B: Each treatment duration: 1L mCRPC treatment (Days)**

| 1L Treatment | N (%) | Mean (SD) | Max | Q3 | Median | Q1 | Min |
|---|---|---|---|---|---|---|---|
| Total | 4967 (100.0) | 406.3 (424.4) | 2841 | 535.0 | 253.0 | 125.0 | 1 |
| Vintage hormone therapy | 247 (5.0) | 311.0 (334.5) | 1719 | 413.0 | 201.0 | 84.0 | 5 |
| Abiraterone | 1404 (28.3) | 427.0 (433.9) | 2731 | 574.0 | 280.0 | 115.0 | 1 |
| Enzalutamide | 2142 (43.1) | 506.2 (473.9) | 2841 | 694.0 | 343.5 | 175.0 | 2 |
| Apalutamide | 94 (1.9) | 397.4 (332.5) | 1317 | 594.0 | 274.5 | 119.0 | 28 |
| Darolutamide | 37 (0.7) | 323.8 (218.0) | 721 | 484.0 | 267.0 | 147.0 | 21 |
| Docetaxel | 532 (10.7) | 231.7 (196.6) | 1701 | 283.5 | 187.0 | 117.5 | 7 |
| Cabazitaxel | 88 (1.8) | 286.2 (304.3) | 2328 | 332.5 | 208.0 | 112.0 | 23 |
| Olaparib | 12 (0.2) | 304.0 (188.2) | 625 | 485.5 | 272.5 | 150.5 | 48 |
| Radium(223Ra) | 215 (4.3) | 159.6 (52.7) | 266 | 196.0 | 174.0 | 147.0 | 28 |
| Abiraterone + Radium(223Ra) | 12 (0.2) | 216.2 (112.2) | 423 | 298.5 | 219.0 | 144.5 | 28 |
| Enzalutamide + Radium(223Ra) | 48 (1.0) | 302.4 (331.0) | 1856 | 299.5 | 196.5 | 153.0 | 6 |
| Pembrolizumab | 1 (0.0) | 98.0 (-) | 98 | 98.0 | 98.0 | 98.0 | 98 |
| Other | 135 (2.7) | 34.5 (58.3) | 484 | 28.0 | 27.0 | 14.0 | 1 |

Aggregated 1L data. 1L mCRPC therapy is the first treatment line initiated after index date.

Notes: Vintage hormone therapy group comprises: treatment with antiandrogens (AA); androgen deprivation therapy (ADT); combined androgen blockade (CAB); or estrogen therapy; mCRPC, metastatic castration-resistant prostate cancer; SD, standard deviation

**Supplementary Data S6: Demographics and baseline characteristics, total population and subgroups**

| **Item** | **Total** (n=4967) | **Subgroup 1 mCSPC to mCRPC** (n=3707) | **Subgroup 2 nmCRPC to mCRPC** (n=1260) |
|---|---|---|---|
| Age at index date 1, years | | | |
| Mean±SD | 74.1±7.8 | 74.0±7.8 | 74.5±7.5 |
| Median (Q1, Q3) | 74.0 (69.0, 80.0) | 74.0 (69.0, 80.0) | 75.0 (70.0, 80.0) |
| Age at index date 2, year | | | |
| Mean±SD | 76.0±7.7 | 75.8±7.8 | 76.5±7.4 |
| Median (Q1, Q3) | 76.0 (71.0, 82.0) | 76.0 (70.0, 82.0) | 77.0 (72.0, 82.0) |
| Age range at index date 2 | | | |
| <=49, n (%) | 8 (0.2) | 8 (0.2) | 0 (0.0) |
| 50-59, n (%) | 107 (2.2) | 79 (2.1) | 28 (2.2) |
| 60-69, n (%) | 858 (17.3) | 694 (18.5) | 174 (13.8) |
| 70-79, n (%) | 2282 (45.9) | 1673 (45.1) | 609 (48.3) |
| 80-89, n (%) | 1575 (31.7) | 1165 (31.4) | 410 (32.5) |
| >=90, n (%) | 137 (2.8) | 98 (2.6) | 39 (3.1) |
| Sex | | | |
| Male, n (%) | 4967 (100.0) | 3907 (100.0) | 1260 (100.0) |
| Female, n (%) | 0 (0.0) | 0 (0.0) | 0 (0.0) |
| Duration from the date of mCRPC to index date 2, days[a] | | | |
| Mean±SD | 6.7±17.6 | 5.8±16.7 | 9.2±19.7 |
| Median (Q1, Q3) | 0.0 (0.0, 0.0) | 0.0 (0.0, 0.0) | 0.0 (0.0, 6.0) |
| Comorbidities | | | |
| Liver disease, n (%) | 721 (14.5) | 554 (14.9) | 167 (13.3) |
| Kidney disease, n (%) | 2101 (42.3) | 1531 (41.3) | 570 (45.2) |
| Cardiovascular disease, n (%) | 1870 (37.6) | 1365 (36.8) | 505 (40.1) |
| Metabolic disease, n (%) | 2572 (51.8) | 1969 (53.1) | 603 (47.9) |
| Prostatic disease, n (%) | 2704 (54.4) | 2024 (54.6) | 680 (54.0) |

[a]The dates of CRPC diagnoses by ICD-10 codes were made on the last day of each month. If 1L or treatment was started in the same month, the duration from mCRPC to index date 2 was calculated as "0".

Notes: mCRPC, metastatic castration-resistant prostate cancer; mCSPC, metastatic castration-sensitive prostate cancer; nmCRPC, non-metastatic castration-resistant prostate cancer; SD, standard deviation; Q1, first quartile; Q3, third quartile

**Supplementary Data S7: Treatment sequence before and after mCRPC (subgroup analysis)**

S7A Subgroup 1

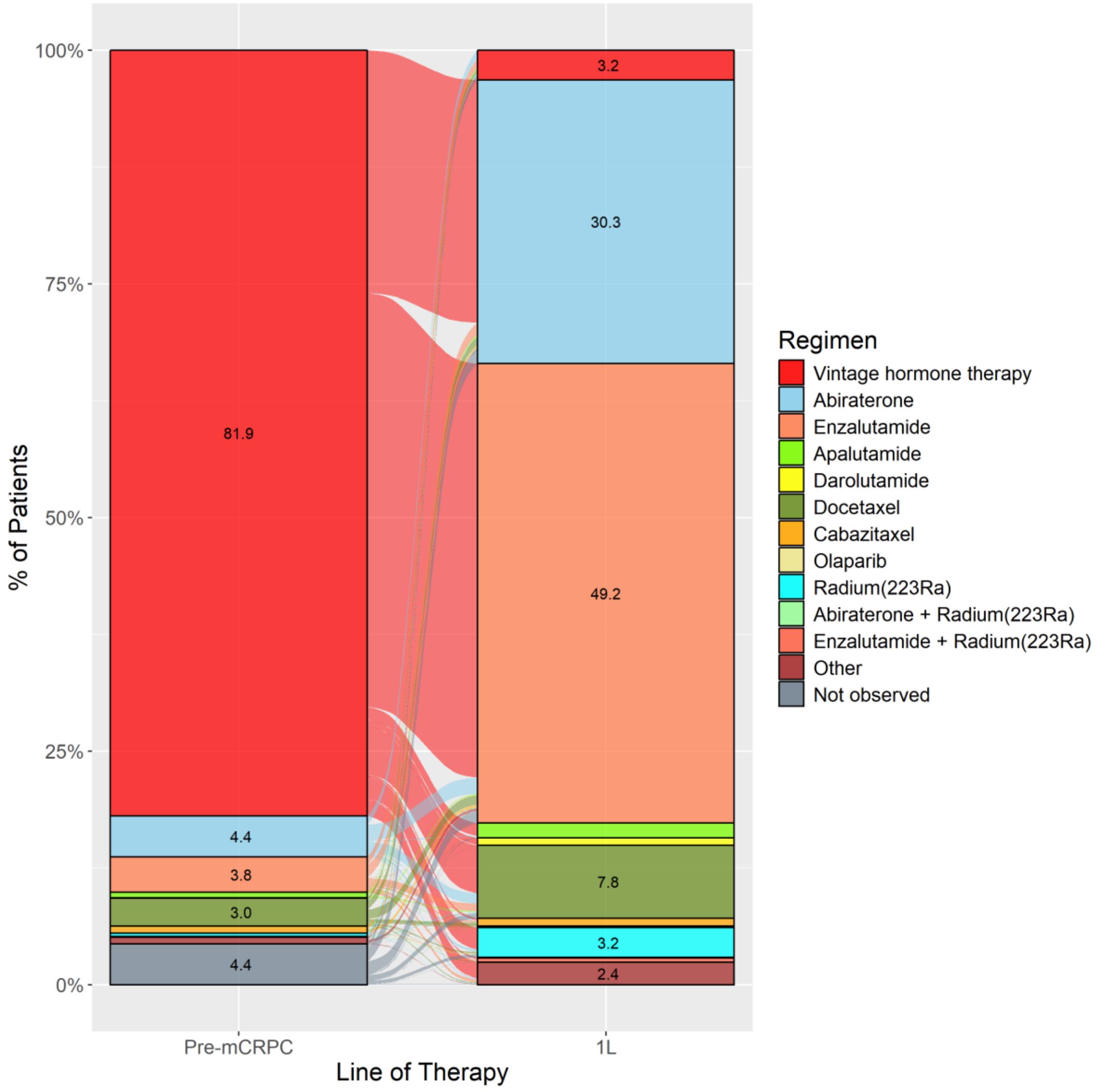


Notes: Vintage hormone therapy group comprises: treatment with antiandrogens (AA); androgen deprivation therapy (ADT); combined androgen blockade (CAB); or estrogen therapy; mCRPC, metastatic castration-resistant prostate cancer.

S7B Subgroup 2

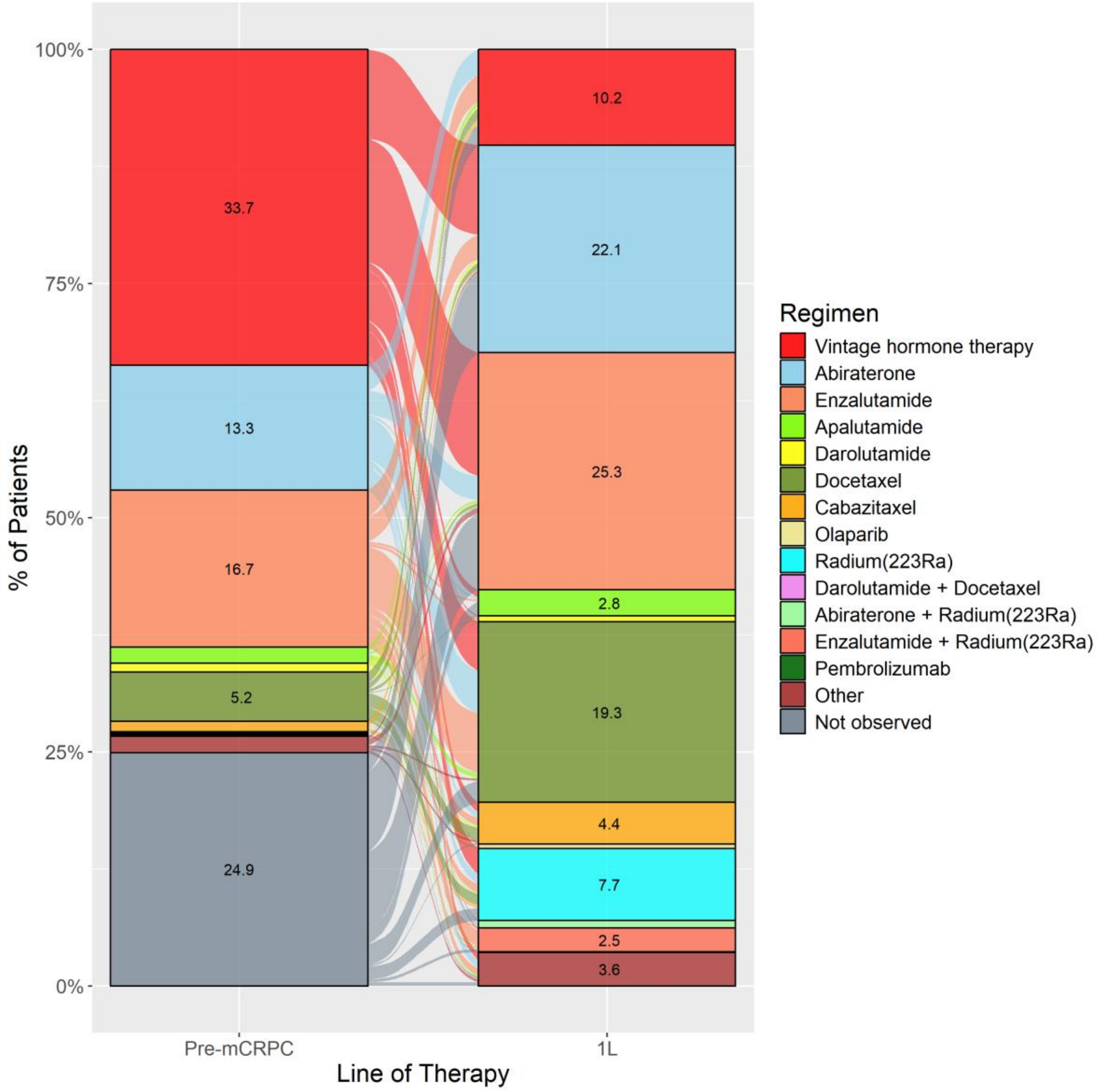


Pre-mCRPC treatment is the last treatment line just before index date. 1L mCRPC therapy is the first treatment line initiated after index date.

Notes: Vintage hormone therapy group comprises: treatment with antiandrogens (AA); androgen deprivation therapy (ADT); combined androgen blockade (CAB); or estrogen therapy; mCRPC, metastatic castration-resistant prostate cancer.

**Supplementary Data S8: Annual trends of pre- and 1L-mCRPC treatment (subgroup analysis)**

S8A: Pre-mCRPC treatment (Subgroup 1)

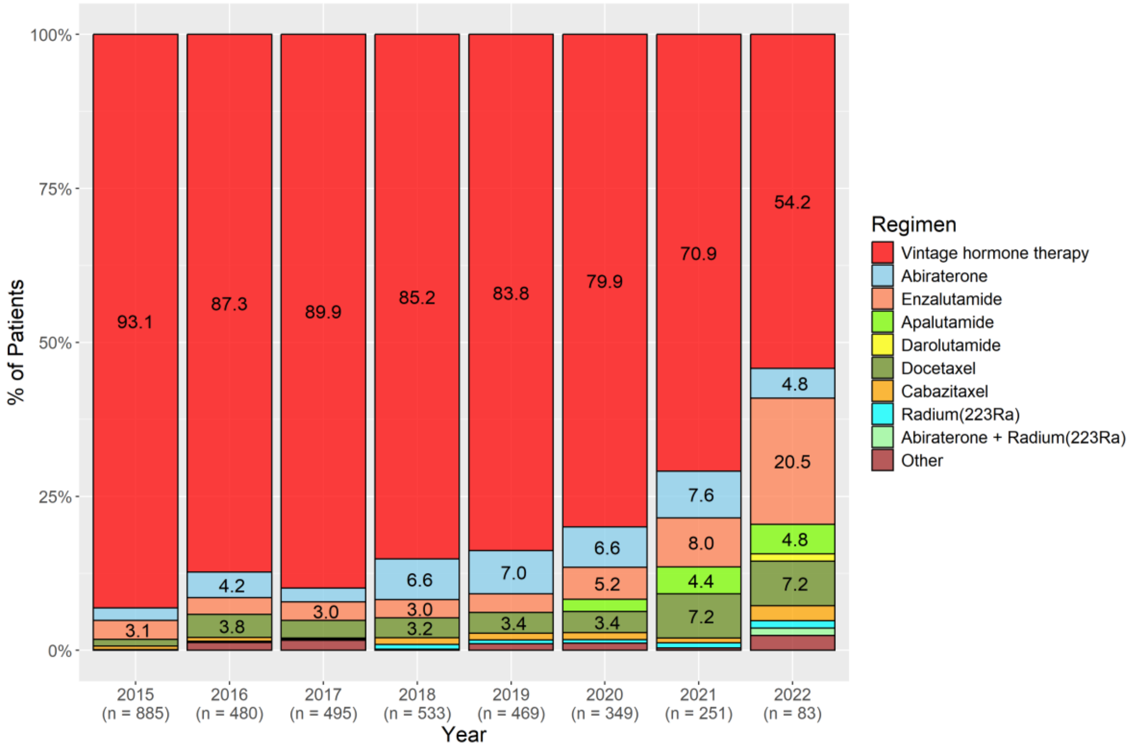


Pre-mCRPC treatment is the last treatment line just before index date.

Notes: Vintage hormone therapy group comprises: treatment with antiandrogens (AA); androgen deprivation therapy (ADT); combined androgen blockade (CAB); or estrogen therapy; mCRPC, metastatic castration-resistant prostate cancer.

S8B: 1L mCRPC treatment (subgroup 1)

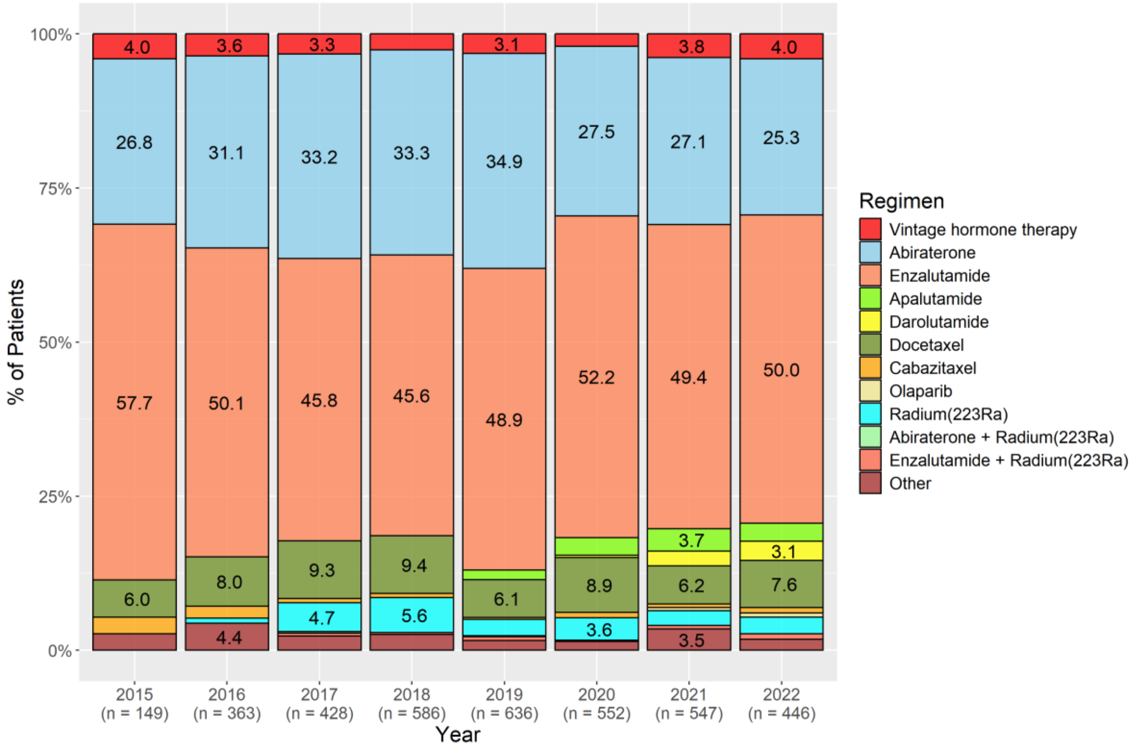


1L mCRPC therapy is the first treatment line initiated after index date.

Notes: Vintage hormone therapy group comprises: treatment with antiandrogens (AA); androgen deprivation therapy (ADT); combined androgen blockade (CAB); or estrogen therapy; mCRPC, metastatic castration-resistant prostate cancer.

S8C: Pre-mCRPC treatment (Subgroup 2)

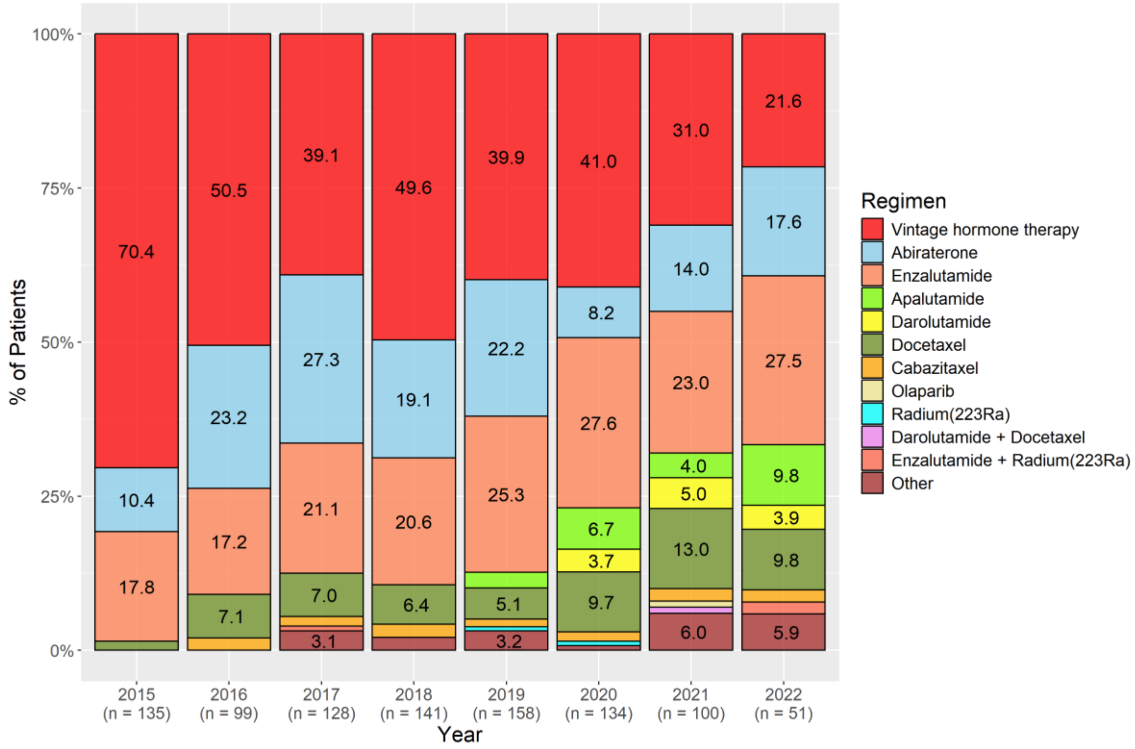


Pre-mCRPC treatment is the last treatment line just before index date.

Notes: Vintage hormone therapy group comprises: treatment with antiandrogens (AA); androgen deprivation therapy (ADT); combined androgen blockade (CAB); or estrogen therapy; mCRPC, metastatic castration-resistant prostate cancer.

S8D: 1L mCRPC treatment (subgroup 2)

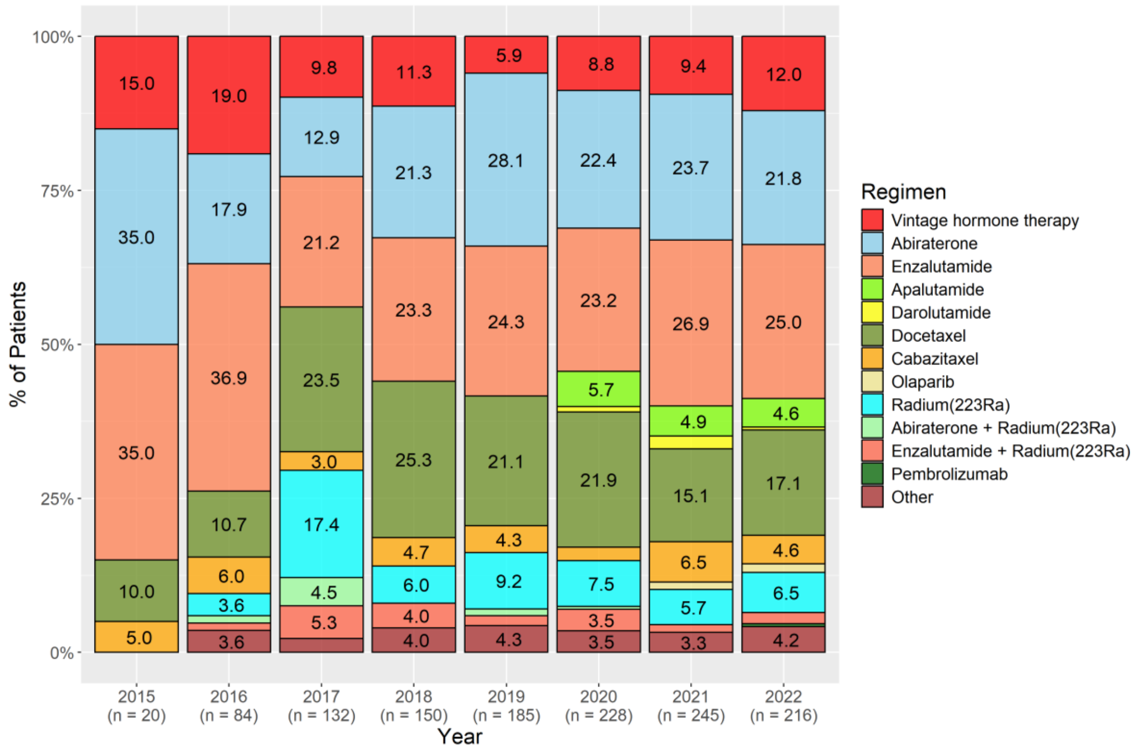


1L mCRPC therapy is the first treatment line initiated after index date.

Notes: Vintage hormone therapy group comprises: treatment with antiandrogens (AA); androgen deprivation therapy (ADT); combined androgen blockade (CAB); or estrogen therapy; mCRPC, metastatic castration-resistant prostate cancer.
